# Selective In Vitro and Ex Vivo Staining of Brain Neurofibrillary Tangles and Amyloid Plaques by Novel Ethylene Ethynylene-Based Optical Sensors

**DOI:** 10.3390/bios13020151

**Published:** 2023-01-18

**Authors:** Florencia A. Monge, Adeline M. Fanni, Patrick L. Donabedian, Jonathan Hulse, Nicole M. Maphis, Shanya Jiang, Tia N. Donaldson, Benjamin J. Clark, David G. Whitten, Kiran Bhaskar, Eva Y. Chi

**Affiliations:** 1 Biomedical Engineering Graduate Program, University of New Mexico, Albuquerque, NM 87131, USA; 2 Center for Biomedical Engineering, University of New Mexico, Albuquerque, NM 87131, USA; 3 Nanoscience and Microsystems Engineering Graduate Program, University of New Mexico, Albuquerque, NM 87131, USA; 4 Molecular Genetics and Microbiology, University of New Mexico, Albuquerque, NM 87131, USA; 5 Department of Neuroscience, University of New Mexico, Albuquerque, NM 87131, USA; 6 Sartorius, Bohemia, NY 11716, USA; 7 Department of Psychology, University of New Mexico, Albuquerque, NM 87131, USA; 8 Department of Chemical and Biological Engineering, University of New Mexico, Albuquerque, NM 87131, USA

**Keywords:** biosensors, neurofibrillary tangles, amyloid plaques, dyes, optical imaging, fluorescence imaging, tau, paired helical filaments

## Abstract

The identification of protein aggregates as biomarkers for neurodegeneration is an area of interest for disease diagnosis and treatment development. In this work, we present novel super luminescent conjugated polyelectrolyte molecules as ex vivo sensors for tau-paired helical filaments (PHFs) and amyloid-β (Aβ) plaques. We evaluated the use of two oligo-p-phenylene ethynylenes (OPEs), anionic OPE_1_^2−^ and cationic OPE_2_^4+^, as stains for fibrillar protein pathology in brain sections of transgenic mouse (rTg4510) and rat (TgF344-AD) models of Alzheimer’s disease (AD) tauopathy, and post-mortem brain sections from human frontotemporal dementia (FTD). OPE_1_^2−^ displayed selectivity for PHFs in fluorimetry assays and strong staining of neurofibrillary tangles (NFTs) in mouse and human brain tissue sections, while OPE_2_^4+^ stained both NFTs and Aβ plaques. Both OPEs stained the brain sections with limited background or non-specific staining. This novel family of sensors outperformed the gold-standard dye Thioflavin T in sensing capacities and co-stained with conventional phosphorylated tau (AT180) and Aβ (4G8) antibodies. As the OPEs readily bind protein amyloids in vitro and ex vivo, they are selective and rapid tools for identifying proteopathic inclusions relevant to AD. Such OPEs can be useful in understanding pathogenesis and in creating in vivo diagnostically relevant detection tools for neurodegenerative diseases.

## 1. Introduction

Proteinaceous deposits composed of β-sheet rich fibrils are pathological hallmarks of a range of neurodegenerative disorders, including Alzheimer’s (AD) and Parkinson’s diseases (PD). Following DNA translation, proteins normally fold into their functional conformations driven by thermodynamics and with the help of chaperone proteins. Many proteins undergo a poorly understood misfolding and aggregation process to form toxic oligomers and then β-sheet rich fibrils, which eventually deposit into large inclusion bodies [1,2,3,4]. Such is the case for the amyloid-β peptide (Aβ) and the tau protein, deposits of which are found in the brains of AD patients.

The Aβ peptide varies in length from 36–42 amino acids and is cleaved from the amyloid precursor protein by β-secretase followed by γ-secretase. Aβ’s function in the brain is still unclear, but the excessive presence of Aβ or hyperphosphorylated/aggregated tau could be a metabolic by-product with gain-of-toxic function effects or an antimicrobial peptide/modifications evolutionarily derived for innate immunity [5]. Aβ can misfold and aggregate extracellularly to form senile plaques, which are key features for AD diagnosis. The tau protein is expressed in the central nervous system and promotes microtubule assembly and stability. There are six tau isoforms, ranging from 352–441 amino acids long, formed by alternative splicing of the *MAPT* gene [4]. Tau’s instability is related to the irreversible hyperphosphorylation by many kinases [6,7], which increases the non-microtubule bound population of tau and results in its intracellular aggregation to form neurofibrillary tangles (NFTs). In addition to AD, NFTs have been found in a range of tauopathies, including frontal temporal dementia (FTD), PD, and traumatic brain injury [8,9,10].

While many factors may contribute to AD etiology [4,5], including inflammation [11,12,13,14] and microbial infection [6,15,16], the misfolding and aggregation of Aβ and tau into fibrillar amyloid aggregates is still recognized as a central pathogenic event and one that is believed to occur decades before the onset of neurodegeneration and cognitive impairment [1,17,18,19]. In fact, a few years ago, the National Institute on Aging (NIA) and Alzheimer’s Association (AA) defined AD diagnostic criteria solely based on the presence or absence of Aβ and tau pathologies as well as neurodegeneration (A+T+N+) [20]. Monitoring the course of amyloid formation at the biochemical, cellular, and tissue levels is thus vital to understanding and combating these diseases [21].

The detection of amyloid protein fibrils has traditionally been carried out using Thioflavin T (ThT) and Congo Red stains [19,22,23]. These dyes consist of a rigid rod-like, conjugated hydrophobic backbone that planarizes (molecular-rotor motif) upon binding to β-sheet rich fibrils, which produce a shift in fluorescence [24,25,26]. Although not used in vivo, these dyes have served as a springboard for a new generation of diagnostic positron emission tomography (PET) radio-ligands for visualizing Aβ plaques in the brain, including Pittsburgh Compound B, ^18^F-florbetapir, ^18^F-florbetaben, and ^18^F-flutemetamol [27,28,29]. The use of these probes to diagnose AD is still limited, partly because they do not detect NFTs. Developing ligands for NFT imaging is an active area of research, and in vivo imaging agents that have been studied include quinoline and benzimidazole derivatives, arquiline derivatives, pyrido-indole derivative AV-1451, and the phenyl/pyridinyl-butadienyl-benzothiazole/benzothiazolium-BB3 derivatives [30,31,32,33,34]. Recently, the FDA-approved ^18^F-T807 (also known as ^18^F-AV-1451, ^18^F-flortaucipir, or TAUVID) showed promise as a cortical tau tracer in AD patients but not in non-AD tauopathies. [35] As NFT burden is more closely linked to the cognitive decline and disease prognosis in AD patients than Aβ plaque load or empirical symptom observations [36,37,38], there continues to be a need to develop robust NFT sensing agents to aid in the tracking of disease progression and correlating it with cognitive decline and brain atrophy.

The development of protein aggregate sensors includes the synthesis and characterization of various classes of compounds based on their biophysical properties. Luminescent conjugated oligo-and polythiophenes (LCOs, 20–23 LCPs, 24–26 and bi-thiophene-vinyl-benzothiazoles bTVBTs 27) have been introduced for broad amyloid identification [11,12,13,39]. Despite this, existing diagnostic tools are too specific and lack the sensitivity required to detect different types of protein aggregates, which are widespread in the most common types of mixed dementia. In an attempt to develop more clinically relevant sensors, we previously tested a small library of synthetic polyelectrolytes, oligo-*p*-phenylene ethynylenes (OPEs) ranging in size and photophysical properties for their specificity and affinity towards fibrillar aggregates of two model amyloid proteins, insulin and lysozyme [14], and disease-relevant Aβ40, Aβ42, and α-synuclein proteins [40]. OPEs possess many desirable properties for detecting amyloids, such as a linear and highly conjugated morphology and several well-characterized fluorescence sensing properties [41]. In addition to the molecular-rotor motif of optical sensing, OPEs also offer additional sensing modes that give rise to distinct fluorescence signatures, including J aggregate formation that results in bathochromic absorbance shifts, sharpened florescence peaks, enhanced fluorescence yield, and narrowed Stokes shifts [42]. Our studies showed that OPEs are superior sensors of fibrils and pre-fibrillar aggregates in vitro compared to ThT [40]. OPEs and ThT share the conjugated backbone with rigid rod architecture, yet OPEs also have tailorable end groups and charged side chains and can vary in length [14]. All these features have been found to be important in OPE’s water solubility, photophysical properties, and affinity and selectivity for aggregated protein conformations [14,15,16,21]. Results from simulation studies showed that OPEs bind to protein assemblies such as viral capsids [43] and Aβ40 protofibrils [44] at multiple sites. Binding free energy is dominated by strong van der Waals interactions between the hydrophobic OPE backbone and hydrophobic grooves and patches on the protein surface and favorable electrostatic interactions between the charged moieties on the OPEs and oppositely charged residues around the binding sites on the protein assemblies. Both anionic and cationic OPEs exhibit favorable interactions with Aβ oligomers [44]. Therefore, OPEs are viable as broad protein aggregate sensors and have diagnostic potential against AD and related proteinopathies. Here we report the screening and identification of novel OPEs as sensors of tau and Aβ aggregates ex vivo. We characterized their sensing of human and mouse brain-derived PHFs and ex vivo staining of brain sections from transgenic mouse (rTg4510) and rat (Tg344-AD) models of AD, as well as post-mortem brain sections from human FTD-tauopathy.

## 2. Materials and Methods

### 2.1. Animals

All experimental protocols involving animals were performed in accordance with US National Institutes of Health guidelines on animal care and were approved (16-200428-B-HSC; 15-200352-HSC; 21-201103-MC) by the University of New Mexico Institutional Animal Care and Use Committee. Animals of both sexes were used in this study.

### 2.2. Dyes

OPE_1_^2−^ and OPE_2_^4+^ were synthesized as previously reported [15] and solubilized in water. The water used was purified to a resistivity of 18.2 MΩ at 25 °C (Millipore Synergy UV purification system, EMD Millipore, Billerica, MA). OPE concentrations were determined by measuring their absorbance values (PerkinElmer Lambda 35 UV/Visible Spectrophotometer); extinction coefficients (ε) of the OPEs in water are ε_370nm_ = 3.92 × 10^4^ M^–1^ cm^–1^ for OPE_1_^2−^ and ε_370nm_ = 8.29 × 10^4^ M^−1^ cm^−1^ for OPE_2_^4+^. ThT was purchased from Thermo Fisher Scientific (Waltham, MA). To prepare the ThT stock solution, ThT powder was solubilized in 70% ethanol in water at 0.5% *w/v* (1.56 mM). All dye solutions were covered with aluminum foil and stored in the dark at 4 °C until use.

### 2.3. Tau441 Monomers and PHF Extraction and Purification

The recombinant tau-441 (2N4R-Tau) monomers were generously provided by Dr. Rakez Kayed, and prepared as previously described [45]. A protein solution at 6.67 μg/mL was prepared by diluting in 10 mM phosphate buffer at pH 7.4. Mouse and human paired helical filaments (PHFs) were extracted using the Sarkosyl extraction protocol at room temperature and briefly described here. Mouse or human brain tissue was first homogenized in 10 volumes of cold buffer H (10 mM Tris-HCl, 1 mM EGTA, 0.8 mM NaCl, and 10 *w/v*% sucrose at pH 7.4). After sonication and centrifugation at 22,000× *g* for 30 min at 4 °C, the supernatant was adjusted to 1 *w/v*% N-laurylsarcosine and 1% β-mercaptoethanol and incubated for 2 h at 37 °C with agitation. Next, the solution was centrifuged at 150,000× *g* for 35 min. The pellet containing the Sarkosyl-insoluble fraction was washed multiple times in 1% buffer H. Then, the pellet was resuspended in Buffer H with 1 *w/v*% CHAPS and 1 *v/v*% β-mercaptoethanol and filtered with a 0.45 µm cellulose acetate syringe filter. This filtered solution was centrifuged at 35,000× *g* rpm for 1 h at room temperature. The pellet was then collected and resuspended in buffer H with 1 *v/v*% β-mercaptoethanol and then purified by layering over a sucrose gradient and centrifuging at 35,000× *g* rpm for 2 h in a Beckman SW 41 Ti rotor at 4 °C. The sucrose gradient was composed of 6 mL of 50% sucrose and 4 mL of 35% sucrose in buffer (10 mM Tris, 0.8 mM NaCl, 1 mM EGTA, and 0.1 *v/v*% β-mercaptoethanol at pH 7.4). Following centrifugation, PHFs were collected from the 35–50% sucrose layer interface with a syringe. Further PHF purification was performed using chloroform/methanol precipitation. First, a 4:1 methanol to protein (*v/v*) solution was prepared and vortexed, then a volume of chloroform was added. The sample was vortexed and then centrifuged at 10,000× *g* for 5 min. The top methanol layer was removed, four more volumes of methanol were added, and the solution was vortexed and centrifuged again at 10,000× *g* for 15 min. The supernatant was removed. The remaining pellet containing PHFs was air-dried and resuspended with cell media. Protein concentration was determined using Nanodrop (Thermo Fisher Scientific, Waltham, MA, USA).

Western blot assay was carried out to confirm that the fraction isolated from the extraction comprised of the tau protein. Sample separation was performed via a 4–12% Bis-Tris Novex NuPage gel from Invitrogen, then transferred to a polyvinylidene difluoride (PDVF) membrane. The membrane was blocked with 5% milk and then incubated with Tau12 antibody from Millipore at 1:10,000 dilution overnight, followed by goat-anti mouse secondary antibody from Jackson ImmunoResearch. For visualization, the membranes were developed using the ECL reagent from Thermo Fisher Scientific with a 5-min exposure. The immunoreactive bands were quantified using Image J [46,47].

### 2.4. Electron Microscopy

The Sarkosyl insoluble pellets were imaged via transmission electron microscopy (TEM) as previously described [48]. Briefly, a pellet was pipetted onto carbon-coated TEM grids and incubated for 5 min at room temperature. The grids were then negatively stained with 2% uranyl acetate for 2 min. Samples were imaged using a Hitachi H7500 (Hitachi, Tokyo, Japan) transmission electron microscope equipped with an Advanced Microscopy Sciences XR60 camera (Advanced Microscopy Techniques, Woburn, MA, USA).

### 2.5. MTT Cell Viability Assay

Neuroblastoma (N2a, ATCC^®^, Manassas, VA, USA) cells were grown in Dulbecco’s Minimum Essential Medium (DMEM, ATCC^®^) supplemented with 10% Fetal Bovine Serum (FBS) and 100X Antibiotic-Antimycotic (Thermo Fisher Scientific). The N2a cells were passaged and incubated at 37 °C with 5% CO_2_. For the toxicity assay, the cells were plated on 96-well plates at 32,000 cells/well and incubated over night for cell attachment. To add the dyes to the cells, the cell media was first replaced with 10% FBS in DMEM with OPE or ThT at concentrations used for staining. Cell media solution containing 10% ethanol was used as a negative control. All samples were incubated for 24 h. To measure cell viability, cell media was replaced with 10% FBS in DMEM and MTT (Promega, Madison WI, USA). The plates were incubated for 4 h before taking absorbance readings at 490 nm using a SpectraMax M2e plate reader spectrophotometer. Five samples were prepared and measured for each experimental condition, and three experiments were run for all conditions. Means and standard error of means (SEMs) of cell viability for each condition were calculated and reported.

### 2.6. In Vitro OPE Sensing

In vitro OPE sensing of tau-441 (2N4R-Tau) monomers and brain-extracted PHFs was evaluated by measuring the fluorescence of the OPEs (1.6 μM) when mixed with each protein sample (6.67 μg/mL) using a PTI QuantaMaster 40 spectrofluorometer. OPE_1_^2−^ and OPE_2_^4+^ excitation wavelengths were 360 nm and 370 nm, respectively, and their fluorescence emission intensities were measured at 450 nm and 460 nm. Spectra of the OPEs alone in 10 mM phosphate buffer at pH 7.4 were also collected.

### 2.7. Brain Tissue Section Preparation

Tissue from multiple transgenic 9-month-old rTg4510, 9-month-old non-transgenic C57BL/6J mice, and 6-month-old non-transgenic C57BL/6J mice were obtained. Following anaesthetization, mice were transcardially perfused with PBS at pH 7.4. The brains were removed, and the left hemispheres were taken for immunochemical assays. After fixing by immersion in 4% paraformaldehyde (PFA), the brains were then placed into a cryoprotection solution (20 *v/v*% glycerol, 20% 0.4 M Sorenson’s buffer) prior to sectioning. A day before sectioning, the brains were transferred to a cryostorage solution (20 mM phosphate buffer with 1 *w/v*% polyviniylpyrrolidone-40, 30 *w/v*% sucrose, and 30% ethylene glycol). To section, the brains were first placed on a flat surface made of frozen 30% sucrose solution and then flash-frozen using dry ice and ethanol. The frozen brains were then placed on the stage of a Leica Sliding Knife Microtome 2010R (Leica Biosystems, Deer Park, IL, USA) and cut into 30 μm thick sagittal sections. Brain sections were placed into cryostorage solutions and kept at −20 °C until immunohistochemical or fluorescence staining was performed. The sections were washed three times with PBS, placed on a shaker, and blocked with a standard blocking solution (5% natural goat serum in 1xPBS-0.4% Triton X (EMD Millipore Burlington, MA)) for one hour prior to staining.

### 2.8. Ex Vivo OPE and Thioflavin T Staining and Imaging

Prior to staining, brain sections were washed in 1xPBS pH 7.4 to remove residual cryostorage solution, then blocked with 5% normal goat serum in 1xPBS-0.4% Triton X for one hour at room temperature with rotation. To stain the brain sections with OPEs, each OPE was diluted from the stock solutions to 5 μM or 0.5 μM using 1xPB at pH 7.2. The brain sections were then immediately immersed and incubated in a staining solution for an hour at room temperature with rotation; samples were covered with foil to reduce light exposure at all times. The sections were then washed 3 times at 5 min each with PBS to remove excess dye. OPE-stained sections were then stained with 1% Sudan Black B (*w/v*% in 70% ethanol) for five minutes at room temperature with rotation, followed by a 1-min wash in 70% ethanol and five 5-min washes with 1xPBS. To stain the brain sections with ThT, the dye was solubilized in 50% ethanol to a final concentration of 0.05 *w/v*%. Sections were incubated with ThT for 8 min in the dark, followed by two 10-s 80% ethanol washes and then 3 water washes. Following all staining treatments, the sections were mounted using VECTASHIELD HardSet Antifade Mounting Medium without DAPI (Vector Labs, Burlingame, CA, USA) and hardened for at least 24 h at 4 °C before imaging.

### 2.9. Ex vivo Immunohistochemical Staining

Primary antibodies, AT180 or 4G8, and the secondary antibody Alexa Fluor 555, were purchased from Thermo Fisher and used without further purification. From their stock solutions (2 mg/mL), AT180 and 4G8 were used at a 1:500 dilution, and AF555 was used at a 1:1000 dilution. Sections were incubated with AT180 or 4G8 overnight at 4 °C; secondary antibody incubation was done for one hour at room temperature in the dark. Following incubation, the excess dye was removed by PBS washes. To block lipofuscin auto fluorescence signals, the sections were treated with 1% Sudan Black B (Sigma Aldrich, St. Louis. MI, USA) for 5 min followed by a 1-min rinse with 70% ethanol, then five 5-min rinses with PBS. Prior to staining, the sections were washed with PBS to remove any residual cryostorage solution and then blocked with 5% normal goat serum in 1xPBS-0.4% Triton X for 1 h at room temperature. Following all staining treatments, the sections were mounted using VECTASHIELD HardSet Antifade Mounting Medium without DAPI and hardened at 4 °C for at least 24 h before imaging.

For rat sections, we used brain sections from 10-month-old transgenic TgF344-AD and non-Tg Fisher-344 rats. The 30 μm brain sections were prepared using on the same protocol as described previously. Free-floating sections were incubated with 5 μM OPE, 0.05 *w/v*% ThT or 500x diluted 4G8 anti-Aβ antibodies. In these animals, we visualized the pathology in the hippocampus, which is affected in AD [49].

### 2.10. Confocal Microscopy Imaging and Quantitative Analysis

Images were taken using a Leica TCS SP8 confocal microscope equipped with 405 nm diode laser line, white light laser, multi-argon lines, and Hybrid Spectral Detectors. OPE_1_^2−^ and OPE_2_^4+^ stained samples were excited using the 405 nm laser line, with emission collected at 420–600 nm or 440–650 nm, respectively. ThT-stained samples were excited using the 458 nm argon line, with their emission collected from 460–650 nm. Sections co-stained with anti-phospho tau antibodies (AT180) or Aβ-specific (4G8) and Alexa Fluor 555 were excited using a tunable and pulsed white light laser (470–670 nm), and emissions were collected from 560–700 nm. All sections were initially imaged using a 20x objective with immersion oil to identify anatomy and then by the 63x objective to collect images. Emission intensities were also taken at 10–20 nm intervals to generate emission spectra. Ex vivo OPE-stained brain tissue images shown in Figure S2 were taken using a Zeiss LSM 900 confocal microscope equipped with a 405 nm solid-state laser line with emission collected from 400 to 600 nm.

To quantify OPE_1_^2−^ and AT180 staining of NFTs, single-stained images from wildtype and transgenic mouse brains in the cortex and hippocampus were analyzed. For each analysis, 3 images were taken per sagittal section, 3 sections were taken from each animal, and 3 animals were used. Thus, a total of 27 images were analyzed for each staining condition. All images were converted to 8-bit, and a threshold was applied to subtract the background signal or remaining lipofuscin fluorescence. To obtain positive staining area in each image, Fiji’s measure tool [47,50] was used to calculate the percentage of positive pixels over the 246 × 246 µm^2^ image area. Average percent positive area values and their associated standard errors of the mean (SEM) were reported.

A similar approach was used to quantify 4G8 and OPE staining of Aβ plaques in rat sections. Images (246 × 246 µm^2^) from the hippocampus of single-stained wildtype and transgenic rats were analyzed. For each analysis, the number of plaques was counted in three areas within the hippocampus, 3 sections were taken from each rat, and 3 rats were used. In single-stained sections (4G8 or OPE_2_^4+^), the plaques were easily visualized. Thus, they were counted without specific signals or size cut-offs. A plaque was counted when the staining pattern and size from the 4G8 antibody and OPE_2_^4+^ dye corresponded to each other.

## 3. Results and Discussion

### 3.1. OPEs Are Selective Sensors of Brain-Derived PHFs In Vitro

**Figure 1:**
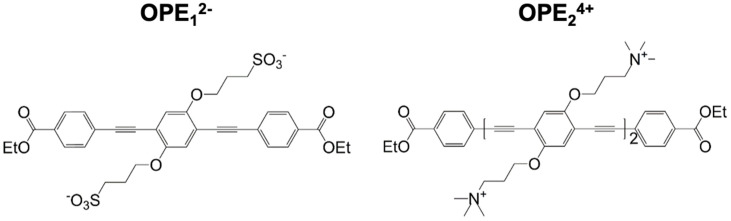
Molecular structures of anionic OPE_1_^2−^ (**left**) and cationic OPE_2_^4+^ (**right**) sensors used in this study. OPE_1_^2−^ has a single repeat unit and sulfonate groups on its side chains. OPE_2_^4+^ has two repeat units and quaternary ammonium groups on its side chains. Both sensors have a conjugated phenylene ethynylene backbone and carboxy-ester end groups. These chemical moieties all play important roles in the detection capability and fluorescence signatures of the sensors.

We had previously screened a small library of 18 conjugated phenylene ethynylene (PPE)-based oligomers (OPEs) and polymers (CPEs) for the detection of fibrillar aggregates of two model amyloid proteins, insulin, and lysozyme [14]. These two proteins differ in size, native structures, and charge at physiological pH but form structurally and morphologically indistinguishable fibrillar aggregates from those formed from disease-associated proteins. Integrated fluorescence intensities of the PPE compounds were quantified in the absence and presence of either monomeric or fibrillar proteins. We found that two small OPEs, the cationic OPE_2_^4+^, and the anionic OPE_1_^2−^ (Figure 1), bound to the amyloid conformation of both insulin and lysozyme and exhibited strong binding-activated superluminescence [14,41]. OPE_1_^2−^, in particular, also showed selectivity as it did not bind to the monomeric conformers of the two proteins. Key features of the selective detection of the fibrillar conformation include moderate size, negative charge, and substituent groups that provide high microenvironment sensitivity to the fluorescence yield [14,41]. In this study, we characterized the efficacy of the two OPEs to detect in vitro brain-derived tau PHFs and stain ex vivo tau NFTs and Aβ plaques in brain sections from transgenic mouse (rTg4510) and rat (Tg344-AD) models of AD, as well as post-mortem brains from human FTD-tauopathy. Evaluating the OPEs sensing capabilities in more physiologically relevant samples enables the further development of these sensors for clinical applications.

**Figure 2:**
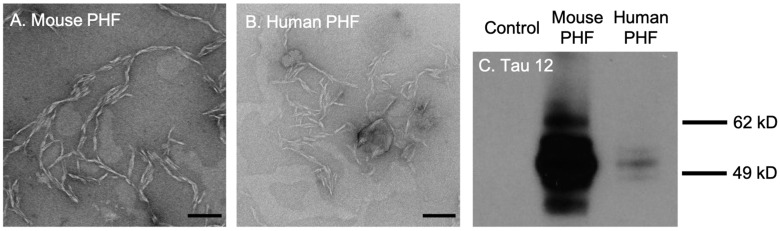
Transmission electron microscopy (TEM) images and western blot analysis of brain-derived paired helical filaments (PHFs). TEM images of PHFs isolated from 9-month rTg4510 mice (**A**) and human patients diagnosed with frontotemporal dementia (**B**). Scale bars = 200 nm. Western blot analysis of brain lysates with the Tau12 antibody (**C**). Note that the bands for mouse PHFs are much darker than human PHFs because the mouse sample was loaded at 10 times the concentration of the human sample.

First, we tested the efficacy and selectivity of cationic OPE_2_^4+^ and anionic OPE_1_^2−^ towards the detection of brain-derived tau PHFs. The PHFs were extracted and isolated from rTg4510 transgenic mice and post-mortem brain tissues from human FTD-tauopathy using a Sarkosyl-insoluble extraction protocol [51]. TEM images of both mouse and human-derived PHFs showed a characteristic twisted fibrillar structure (Figure 2A,B). Western blot assay conducted using the Tau12 antibody, which is specific for the N-terminus of the human tau [52], showed bands between 49 and 62 kD from both mouse and human-extracted PHFs (Figure 2C), confirming that the isolated PHFs were indeed comprised of tau.

We have previously shown that when solubilized in an aqueous buffer, the OPE sensors are quenched and undergo a non-radiative, water-mediated decay process upon excitation by photon absorption [53]. When bound to amyloid fibrils in vitro, through a combination of Coulombic, hydrophobic, and electrophilic/nucleophilic interactions [15], OPEs become highly fluorescent through a number of mechanisms, including restriction to the rotation of the backbone, unquenching of the end groups, and OPE-OPE complex formation at the fibril binding sites [15,53].

**Figure 3:**
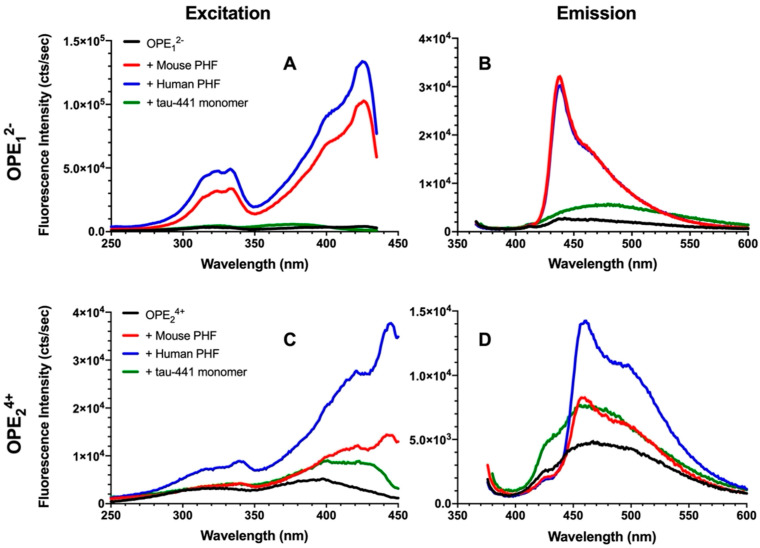
Fluorescence excitation (**A**,**C**) and emission spectra (**B**,**D**) of OPE_1_^2−^ (**A**,**B**) and OPE_2_^4+^ (**C**,**D**). OPE and tau concentrations were 1.6 µM and 6.67 µg/mL, respectively. OPE_1_^2−^ λ_excitation_ = 360 nm and λ_emission_ = 440 nm; OPE_2_^4+^ λ_excitation_ = 370 nm and λ_emission_ = 460 nm.

To evaluate the efficacy and selectivity of OPEs’ detection of brain-isolated PHFs, 1.6 μM OPE sensors were added to 6.67 μg/mL PHFs or tau-441 monomers [54], and excitation and emission spectra of the OPEs were collected (Figure 3). As shown in the emission spectra, OPE_1_^2−^ alone is weakly fluorescent, displaying a small peak at around 440 nm (Figure 3B). When the OPE was added to tau-441 monomers, there was a small increase in OPE fluorescence intensity and a red shift where fluorescence peaked at around 470 nm. Conversely, OPE_1_^2−^ fluorescence intensity significantly increased with a sharp peak at 440 nm and a broader peak at 470 nm when the OPE was incubated with human and mouse brain-derived PHFs (Figure 3B). OPE_1_^2−^ thus selectively detected tissue-derived PHFs over the monomeric tau-441. These sharp and broad spectral peaks likely arise from OPE binding to different sites on the fibril and the formation of OPE complexes [55]. To quantify OPE’s selectivity, we calculated amyloid detection factor (ADF) values using Equation (1) [14]:

(1) $$ADF=\frac{I_{dye+PHF}-I_{dye+monomer}}{I_{dye+monomer}}.$$

where $I_{dye+PHF}$ is the integrated fluorescence intensity of the sample containing dye and PHFs and $I_{dye+monomer}$ is the integrated fluorescence intensity of the sample containing dye and tau-441 monomers. ADF values greater than 1 indicate selective detection of PHFs over monomeric tau, ADF values less than −1 indicate selective detection of monomeric tau-441 over PHFs, and ADF values between −1 and 1 indicate no significant selectivity. The ADF values of OPE_1_^2−^ for mouse and human-derived PHFs were 2.74 and 2.62, respectively (Table 1), indicating that the OPE selectively detected both human and mouse PHFs and the selectivity of the two PHFs were comparable.

Fluorescence of OPE_2_^4+^ similarly increased in the presence of PHFs compared to the quenched sensor alone (Figure 3C,D), but the increase was higher for human PHFs. OPE_2_^4+^ alone displayed a low level of fluorescence, although higher than the single repeat unit OPE_1_^2−^. When added to monomeric tau-441, the shape of the sensor’s fluorescence profile remained the same, but its overall fluorescence intensity increased. When added to the PHF samples, in addition to increases in fluorescence intensity, changes to the spectral shape where a sharp peak at ~460 nm and a broad peak at ~500 nm were observed, these changes are similar to those observed from OPE_1_^2−^. The higher fluorescence increase observed for human-derived PHFs compared to mouse-derived PHFs perhaps reflects subtle differences in their fibril structures. ADF value calculated for mouse PHFs was −0.21, indicating that based on overall fluorescence intensity, OPE_2_^4+^ did not selectively detect mouse PHFs over tau monomers. However, differences in their spectral features could be used to differentiate the two tau conformations, monomers vs. PHFs. ADF value calculated for human PHFs was 0.55, which indicates modest selectivity. Again, the distinct spectral differences of OPEs bound to tau monomers vs. human PHFs could help to differentiate the two conformers of human tau.

Overall, in vitro sensing of tissue-derived PHFs shows that OPE_1_^2−^ selectively detected the PHFs from both human and mouse brains. OPE_2_^4+^ similarly showed fluorescence enhancements in the presence of the PHFs but also displayed a fluorescence increase in the presence of tau monomers. These results are consistent with those obtained from in vitro sensing of lysozyme and insulin fibrils [14,41], wherein OPE_1_^2−^ was a sensitive and selective sensor and OPE_2_^4+^ detected both tau monomers and PHFs.

### 3.2. OPE_1_^2−^ and OPE_2_^4+^ Ex Vivo Detection of NFTs in rTg4510 Mice and Human FTD Tauopathy Brain Sections

To validate OPEs as selective sensors for protein aggregates ex vivo, we stained brain sections from 9-month-old transgenic rTg4510 mice and human subjects with a neuropathological diagnosis of frontotemporal dementia with tauopathy (FTD). The rTg4510 mouse model expresses human *MAPT* that carries the P301L mutation in exon 10, which in humans causes a familial form of tauopathy called frontotemporal dementia, and Parkinsonism linked to chromosome 17 (FTDP-17) [56]. rTg4510 mice display age-related tau hyperphosphorylation and aggregation as PHFs and NFTs in the cortex and hippocampus, neuronal loss, and memory impairments [56,57,58]. Brain sections from 6-months old non-transgenic C57BL/6J (B6) mice were stained as negative controls. Free-floating 30 μm thick brain sections were stained with either 5 μM or 0.5 μM OPEs. Because the OPE dyes were excited at 405 nm, auto-fluorescent features such as lipofuscin were also visible. The brain sections were therefore incubated with 1% Sudan Black B in the last step to block autofluorescence. Another set of sections was also stained with ThT at two concentrations, 5 μM to match the OPEs and 1.57 mM to match published studies [59], where 0.05% *w/v* was used (Supplemental Figure S1). 9-month-old C57BL/6J mice and non-demented healthy human brain tissue sections were also stained with 5 µM and 0.5 µM OPEs as additional negative controls (Supplemental Figure S2).

**Figure 4:**
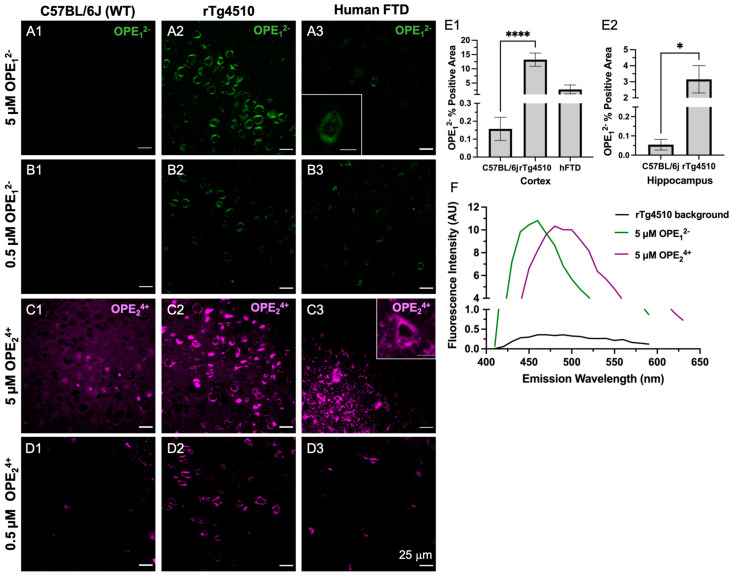
Confocal microscopy images of ex vivo OPE staining of cortex brain sections from non-transgenic C57BL/6J mice (left column), transgenic rTg4510 mice (middle column), and human frontotemporal dementia (right column). At 5 µM (**A1**–**A3**) and 0.5 µM (**B1**–**B3**), OPE_1_^2−^ detected NFTs in rTg4510 mice and human sections and showed no fluorescence in the non-transgenic sections. At 5 µM (**C1**–**C3**), OPE_2_^4+^ stained NFTs in mouse and human sections. However, there was also non-NFT binding and background staining. At 0.5 µM OPE_2_^4+^ (**D1**–**D3**), the background and non-specific staining in mouse and human brain sections were significantly reduced while NFT detection in these sections persisted. Scale bars = 25 μm. Quantification of OPE_1_^2−^ staining (**E1**–**E2**) based on confocal images from the cortex and hippocampus of wildtype and transgenic animals **** *p* < 0.0001, * *p* = 0.01. The emission spectra from unstained (background) and OPE-stained sections (**F**).

As shown by the confocal microscopy images in Figure 4, both OPE_1_^2−^ and OPE_2_^4+^ detected NFTs in transgenic mice brain sections at 5 and 0.5 μM dye concentrations. OPE_1_^2−^ stained NFTs in the cortex with minimal background fluorescence (Figure 4A,B). The signal from NFT-bound OPE appeared peri-nuclear and somato-dendritic with a classical ‘ghost tangle’ pattern, which is characteristic of pathological tau aggregates in the rTg4510 mice [60]. OPE_1_^2−^ also stained NFTs in the human FTD brain sections with minimal background fluorescence at both concentrations tested (Figure 4A3,B3). In contrast, the non-transgenic mouse brain sections did not show any OPE_1_^2−^ staining, as no fluorescent features were observed in these samples (Figure 4A1,B1 and Figure S2A1,B1) nor was there any OPE_1_^2−^ staining in the healthy human brain sections (Figure S2A2,B2). Taken together, our results show that OPE_1_^2−^ stained NFTs-like features in both mouse and human brain sections with little nonspecific staining, indicating that the OPE selectively binds to β-pleated NFTs and exhibited binding-activated fluorescence.

On the other hand, OPE_2_^4+^ at 5 µM prominently stained NFTs in the rTg4510 mice and human FTD brain sections (Figure 4C2,C3). However, more background staining was also observed in the wildtype sections compared to those stained with anionic OPE_1_^2−^. While the exact binding targets other than NFTs for OPE_2_^4+^ are not clear, based on the signal observed in the wildtype mice brain sections, OPE_2_^4+^ may be binding to endogenous mouse tau, nuclear materials, or other smaller amyloidogenic tau aggregates [61]. Lowering the concentration of OPE_2_^4+^ by 10-fold to 0.5 µM significantly reduced background and non-specific staining (Figure 4D1 and Figure S2D1,D2). At this low concentration, OPE_2_^4+^ still stained NFTs in the rTg4510 and human brain sections, but more selectively and with better signal-to-noise ratios (Figure 4D2,D3). As the human sections did not contain the hippocampal area, we only stained the human cortex.

A quantitative analysis was also carried out to compare the staining between wild type and transgenic brain sections from the cortex (Figure 4E1) and hippocampus regions (Figure 4E2). The analysis included setting a threshold to subtract the background fluorescence of single-stained images, followed by using Fiji’s analyze functions to obtain the number of positive pixels in the images. A total of 27 images were analyzed for each staining condition. The averaged percentage of positive pixels and associated errors were reported (Figure 4E1,E2). As shown, percentages of positive areas stained by OPE_1_^2−^ of transgenic mice cortex and hippocampus sections were significantly higher than sections stained from wildtype mice, indicating the successful OPE staining of tau pathology.

To compare the staining performance of OPE_1_^2−^ to the most widely used gold standard amyloid dye ThT, the brain sections from the same animals were stained with 5 μM ThT (Figure S1). At this concentration, no NFTs were detected. Note that ThT staining of 9-month-old rTg4510 and human FTD-tauopathy brain sections have been previously reported [56], but at a much higher concentration of 0.05 *w/v* % [59] or 1.56 mM. This is more than 300 times higher than the 5 μM OPE concentration used in this study. We thus additionally carried out ThT staining at 1.57 mM. As expected [56], ThT yielded robust NFT detection in the Tg4510 mice and human FTD-tauopathy brain sections (Figure S1). For the remainder of the study, all ThT staining was performed at the higher 1.57 mM concentration. Because OPE_2_^4+^ exhibited more non-specific binding than OPE_1_^2−^ in this animal model at 5 μM and OPE_1_^2−^ exhibited robust and selective detection of brain-derived PHFs and staining of NFTs in the mouse and human brain sections, we carried out a co-localization study only with OPE_1_^2−^.

### 3.3. OPE_1_^2−^ Displays a Similar Binding Pattern to Historical Amyloid Stain Thioflavin T

**Figure 5:**
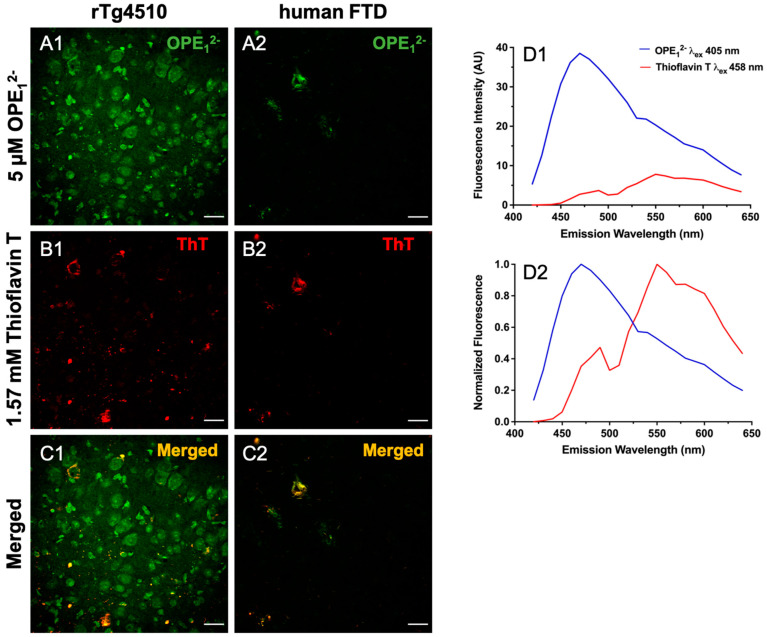
Co-localization of OPE_1_^2−^ and ThT staining in rTg4510 mice and human tissue sections. Scale bars are 25 µm. Tissue was first stained with 5 µM OPE_1_^2−^ and then by 1.57 mM ThT. The OPE_1_^2−^ channel displayed NFT binding in both the mice (**A1**) and the human sections (**A2**), although we note that there was more background staining in the OPE_1_^2−^ channel in co-stained sections than in single-stained sections (Figure 4A2). The ThT channel also displayed NFT binding in both mice (**B1**) and humans (**B2**) at a concentration 300x higher than OPE_1_^2−^. The merged images (**C1**,**C2**) show direct overlap in the human section (**C2**) but more limited overlap in mice (**C1**). OPE_1_^2−^ and ThT emission spectra from the co-stained sections displaying measured fluorescence (**D1**) and normalized fluorescence (**D2**) where signals from the two dyes are reasonably separated and resemble spectra from single-stained sections. Thus bleed-through was unlikely.

In a co-localization study, we used 5 µM OPE_1_^2−^ followed by 1.57 mM ThT on 30 μm free-floating sections from rTg4510 and hFTD brains. Co-localization of these dyes confirms OPE’s staining of and selectivity towards protein aggregates with β-pleated conformation. To visualize co-localization, the two fluorophores were excited and imaged separately, OPE_1_^2−^ excited at 405 nm and ThT excited at 458 nm, and the resulting images were merged to show overlap (Figure 5). We observed partial co-staining in a few sub-fields (Figure 5C1,C2). In the rTg4510 mice brain sections, the OPE_1_^2−^ channel displayed more staining (Figure 5A1) than the ThT channel (Figure 5B1), where OPE_1_^2−^ appeared to label structures that were not detected by ThT. These structures are possibly either oligomeric or pre-tangle forms of tau. Resolving these structures requires further investigation with techniques like laser capture microscopy coupled with mass spectrometry. We also switched the order of staining, i.e., ThT first followed by OPE_1_^2−^, to evaluate if ThT would stain more features, but the same pattern of more staining in the OPE channel was observed. This indicates that OPE_1_^2−^ binds to tissue irrespective of the binding order, possibly with enough binding strength to displace bound ThT if sections were stained with ThT first. In the human FTD brain sections, images from both OPE_1_^2−^ and ThT channels are very similar and almost completely overlap (Figure 5A2,B2). Moreover, signal intensity from the OPE channel was about 4-times higher than that from ThT. Thus, OPE_1_^2−^ performed better than the ThT stain, even at a concentration more than 300 times lower.

Considering the excitation wavelengths and emission spectra of these dyes somewhat overlap, we characterized single-stained sections to ensure the signals we observed were not bleed-through from the neighboring channel. We collected spectra from single-stained sections and compared to those from the co-stained sections’ spectra. Single-stained OPE_1_^2−^ excited at 405 nm has a peak near 460 nm (Figure 4F) and a generally higher fluorescence intensity than single-stained ThT, which has a peak near 555 nm when excited at 458 nm (Figure S1). Emission spectra from the co-stained sections (Figure 5D1,D2) showed that each dye could be excited separately and emit at different wavelengths. We also normalized the signals to better compare the peaks. The signal seen in either channel corresponds to each sensor’s emission and thus was not bleed-through. These results, alongside the in vitro fluorimetry results [62] suggest that OPE_1_^2−^ is a superior sensor compared to ThT with notable higher sensitivity towards NFTs.

### 3.4. OPE_1_^−^ Displays a Similar Binding Pattern to Anti-Phospho Positive Tau Antibody AT180

**Figure 6:**
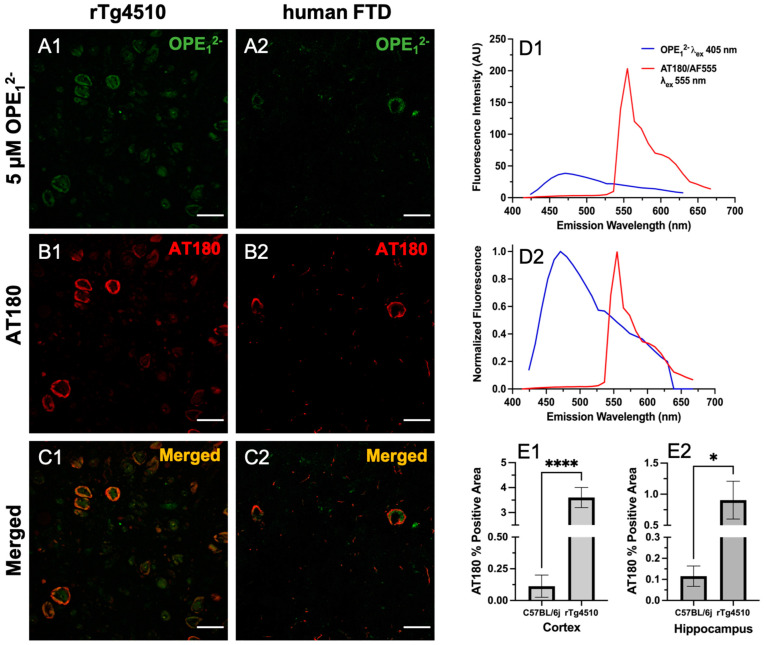
Confocal fluorescence images of sections co-stained with 5 µM OPE_1_^2−^ and anti-phospho tau AT180. Scale bars are 25 µm. The OPE_1_^2−^ channel (**A1**,**A2**) showed NFT pathology in both mice and humans. The anti-phospho tau antibody linked to AlexaFluor 555 (AF555) also stained NFTs in the sections (**B1**,**B2**). The merged images (**C1**,**C2**) show overlap in signals. Some background staining in the OPE_1_^2−^ channel is observed. Measured (**D1**) and normalized (**D2**) emission spectra of OPE and AF555 from the brain sections show that the fluorescence of AF555 is significantly higher. Quantification of AT180 binding in the cortex (**E1**) and hippocampus (**E2**) of transgenic rTg4510 and wildtype C57BL/6J mice (**** *p* < 0.0001, * *p* = 0.01).

To validate OPE’s selectivity towards tau NFTs, we co-stained rTg4510 and hFTD brain sections with OPE_1_^2−^ and the anti-phospho-T231 positive tau antibody AT180. Hyperphosphorylation of threonine 231 (T231) on tau is one of the well-established neuropathological markers of tau pathology [57]. We incubated free-floating brain sections first with 5 µM OPE_1_^2−^ then further processed for AT180 binding and the staining of corresponding anti-mouse secondary antibody conjugated to Alexa Fluor 555 fluorophore (AF555). Each fluorophore was excited separately, and images were combined to visualize for overlap (Figure 6). As shown, there was a striking co-localization of OPE_1_^2−^ with AT180 in the cortical sections of rTg4510 and human FTD brains (Figure 6C1,C2). Structures positive for OPE (Figure 6A1) but negative or less intense for AT180 (Figure 6B1) were observed, which could be PHFs/NFTs where either the AT180 epitope was masked and not accessible or likely positive for other phosphorylated tau antibodies such as AT8 (pS199/pS202/pT202), PHF-1 (pS396/pS404), AT270 (pT181) or other non-AT180 species of tau. It is also possible that these structures are not tau but interact with the OPE, which requires further investigation. In the human FTD brain sections, the AT180 antibody detected two neurons with classical NFTs (Figure 6B2), while OPE_1_^2−^ detected parts of the NFTs (Figure 6B1). When merged, the image (Figure 6C2) shows that the signals did not directly overlap but were slightly juxtaposed. We do not expect complete pixel-by-pixel signal overlap because there are factors still unaccounted for in this experiment and analysis, which will need more research to resolve. For example, there could be competitive binding events, and more characterization of OPE’s non-sequence specificity in tissue staining is needed. The AT180 channel displayed a higher fluorescence intensity (Figure 6D1), likely due to signal amplification from the secondary antibody conjugated to a strong AF555 fluorophore and the AT180 specificity for the T231 phosphorylate tau site. For further and more accurate co-localization analysis and fluorophore optimization, super-resolution imaging could provide more insight.

Based on the co-localization pattern between OPE_1_^2−^ and AT180, we compared OPE_1_^2−^ NFT detection to AT180 by quantifying the percentages of positive pixels in single-stained sections. Again, for each staining condition, a total of 27 images were analyzed, and average percentages of the positive area and associated errors are shown in Figure 6E1,E2. The rTg4510 mice display early NFT formation in the cortex and progressively appears in the hippocampus [56]. By 9 months of age, the animals are severely impaired and have large degrees of neurodegeneration. As shown in Figure 4E1,E2, OPE_1_^2−^ detected significantly higher percentages of positive areas in rTg4510 compared to in C57BL/6J. Similarly, AT180′s percent positive areas were also significantly larger in the cortex and hippocampus (Figure 6E1,E2). Moreover, the percentage of positive areas detected by OPE_1_^2−^ in both the cortex and hippocampus were significantly larger than those by AT180. Overall, this co-localization study confirmed OPE’s staining of tau NFTs and showed that OPE_1_^2−^’s performance was comparable to AT180 in detecting pathology in the transgenic animals in comparison to fluorescent signals in the non-transgenic brain sections.

### 3.5. OPE_2_^4+^ Ex Vivo Staining of TgF344-AD Rats

In previous studies, we found OPEs detected Aβ_40_ and Aβ_42_ fibrils and oligomers in vitro [40]. Binding of OPEs to Aβ fibrils induced fluorescence turn-on and a blue shift in OPE’s spectra compared to its background fluorescence. Here, we tested OPE_1_^2−^ and OPE_2_^4+^ staining capacity for Aβ and tau amyloid pathology in the TgF344-AD transgenic rat model of AD, which expresses amyloid-precursor protein (APP) with Swedish mutant human APP_SW_ and presenilin (PS1ΔE9 mutations) and is in Fisher 344 genetic background [49,63]. This transgenic rat model provides a unique opportunity for research because it presents an animal model with full AD pathological markers, a human-like etiology of the disease mimicking behavioral, morphological, physiological, and genetic aspects [64,65]. While mice models have allowed us to explore AD, a majority of these animals are limited by their lack of presentation of the full spectrum of disease markers, such as neuroinflammation, aggregation of phosphorylated tau, neuronal loss, and time-dependent disease progression, amongst others [66]. The TgF344-AD rats express 2.6-fold higher secretion of APP and 6.2 fold-higher levels of human PS1 compared to endogenous levels [49,63]. Rats express tau with the same six isoforms as in humans, except with slight differences in ratios [8]. The pathology in this model displays Aβ plaques first around six months of age, with the plaque deposition increasing with age to ~10–82-fold by 15–17 months of age based on characterization by 4G8 antibody and Thioflavin S (ThS) staining [63]. Mature NFTs composed of endogenous rat tau are not detected until about 15 months of age in the TgF344-AD rats [49]. Spatial cognitive deficits are observed as early as 7–8 months of age, with definite impairment in spatial navigation at 10–11 months of age based on measurements of swim trajectories in water maze tests [49]. However, significant cognitive declines, robust Aβ pathology, and the appearance of tau burden are not observed until about 15 months of age [64].

**Figure 7:**
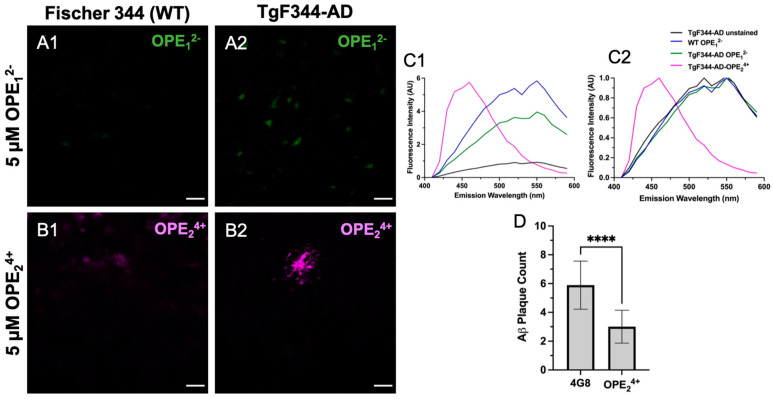
Confocal microscopy images of hippocampus in 10-month-old TgF344-AD rats stained with 5 μM OPE_1_^2−^ (**A1**,**A2**) and OPE_2_^4+^ (**B1**,**B2**). In OPE_1_^2−^ staining, we did not observe typical plaque or NFT staining in the transgenic model, although there appears to be some autofluorescence in the transgenic model (**A2**). In OPE_2_^4+^ staining, a plaque in the TgF344-AD section was clearly visible with minimal background staining. Fluorescence emission spectra, measured (**C1**) and normalized (**C2**), were collected using the LEICA software. The OPE_2_^4+^ stained sections had a spectrum similar to those measured in vitro with a broad emission peak around 450 nm. The spectra collected from OPE_1_^2−^ stained sections were similar to those from the wildtype or unstained sections, indicating a lack of OPE_1_^2−^ binding. Quantitative analysis comparing plaque detection between Aβ-specific antibody 4G8 and OPE_2_^4+^ (**** *p* < 0.0001) (**D**). Scale bars are 25 µm.

At 10 months of age, the TgF344-AD rats did not show hyperphosphorylation of tau at the T231 site as there was a clear lack of AT180 signal (Figure S3), which is consistent with prior studies [63]. Interestingly, the anionic OPE_1_^2−^ had no apparent selective staining of Aβ plaques or any pathology in TgF344-AD rat brain sections, except that it showed some non-specific staining (Figure 7A1,A2). Based on the emission spectra, the fluorescence collected from OPE_1_^2−^ appeared to be autofluorescence in the tissue since it displayed spectral features similar to those from unstained sections, except with slightly higher intensity (Figure 7C1,C2). On the other hand, the cationic OPE_2_^4+^ molecule had better selectivity towards the Aβ plaques (Figure 7B2). Interestingly, at the 5 µM concentration, we did not observe background staining with OPE_2_^4+^ as we did in the mice; even the non-Tg Fisher 344 sections did not show background fluorescence at 5 μM concentration. The spectra of OPE_2_^4+^ stained TgF344-AD brains were similar to those observed with tau and Aβ fibrils in solution in vitro [40].

**Figure 8:**
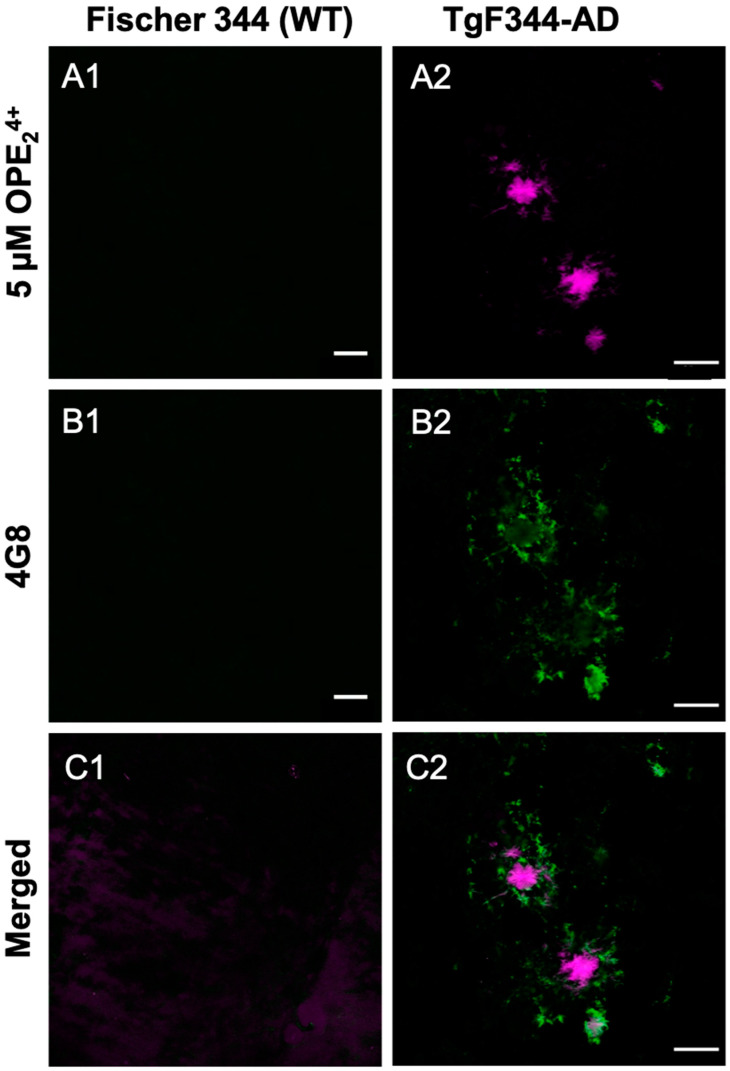
Confocal microscopy images of 10-month-old TgF344-AD rats stained with OPE_2_^4+^ (**A1**, **A2**) fol-lowed by Aβ antibody 4 G8 (**B1**, **B2**) and AF555. The OPE binds strongly to the center of the plaques while the antibody highlights the surrounding loose plaque (**C1**, **C2**). Scale bar 25 µm.

Since the cationic sensor OPE_2_^4+^ detected Aβ plaque pathology in the rat model, we carried out a co-localization study using the 4G8 antibody to validate the pathology specificity staining of our sensor. The 4G8 antibody is specific for the 17–24 amino acid sequence of APP/Aβ. The sections were first stained with OPE, then 4G8, and finally a secondary antibody with Alexa Fluor 555 fluorophore. Each fluorophore was excited separately, and images were combined to visualize overlap (Figure 8). As shown, the OPE_2_^4+^ signal was spatially correlated with the 4G8 signal around Aβ plaques. However, the signals were not directly overlapping where the OPE sensor appeared to bind the dense core of the Aβ plaque while the antibody found its epitope in the diffused plaque areas. The diffuse plaque binding was also found in single-stained 4G8 sections (Figure S3). The dense core plaque is known to be rich in β- sheet fibrils, while diffuse plaques contain more amorphous Aβ deposits [67,68]. As controls, we stained the TgF344-AD and non-Tg rat brain sections with 5 µM and 1.57 mM ThT. We did not observe any staining of Aβ plaque pathology at either ThT concentration (Figure S3).

A quantitative analysis was performed to characterize how effectively OPE_2_^4+^ detects plaques in comparison to the highly specific 4G8 antibodies (Figure 7D). Similar to the quantification protocol used for mice sections, plaques were counted in three areas per section, three sections per rat, and in three rats. This quantification was based on plaque counts in the hippocampus of the rats from sections single-stained with antibody or OPE_2_^4+^ (Figure 7D). Antibody-based detection appeared to be more sensitive than OPE_2_^4+^ in detecting plaques. In TgF344-AD rats, OPE_2_^4+^ bound to the core of Aβ plaques, co-localizing with 4G8 antibody, indicating an affinity for pathologically derived human Aβ protein. We did not detect any AT180 reactive hyperphosphorylated tau or NFTs in 10-month-old TgF344-AD transgenic rats; therefore, we did not test OPE staining of NFTs in rats.

### 3.6. OPEs Are Not Toxic to N2a Neuroblastoma Cells

**Figure 9:**
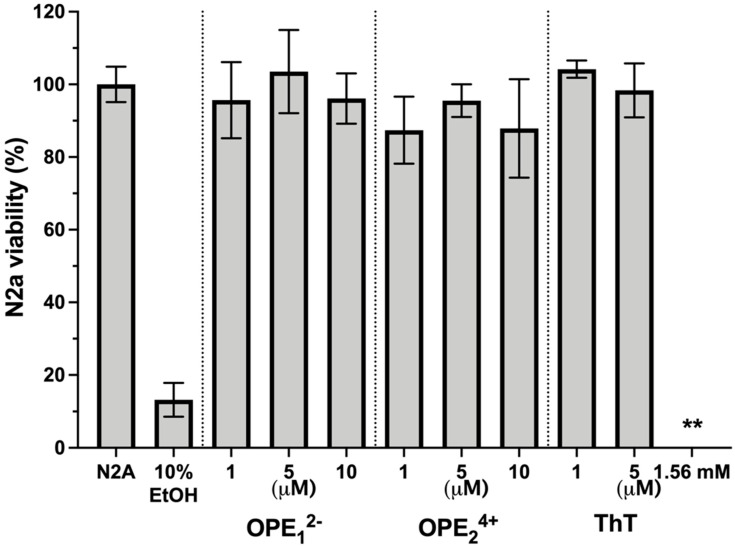
Viability of N2a cells treated with OPEs and ThT at various concentrations as determined by the MTT assay. Controls of untreated cells (N2a) and cells treated with 10% ethanol (EtOH) are also included. Results are normalized to untreated cells (N2a); error bars represent standard errors of means from three experiments. For cells treated with 1.56 mM ThT, no viability was detected.

One potential application of OPEs is the in vivo detection of amyloid protein deposits in the brain. For this application, it is important to determine if these novel phenylene ethynylenes are potentially cytotoxic. We tested OPEs’ toxicity on the mouse N2a neuroblastoma cell line using the 3-(4,5-dimethylthiazol-2-yl)-2,5-diphenyltetrazolium bromide (MTT) cell metabolism assay [69]. Cells were incubated in cell media at 37 °C and 5% CO_2_ with OPE_1_^2−^ or OPE_2_^4+^ at 1 µM, 5 µM, and 10 µM or with ThT at 1 µM, 5 µM, and 1.57 mM for 24 h. Following the incubation, cells were washed with media, and the MTT assay was carried out (Figure 9). All measurements were normalized to the formazan absorbance signal observed from N2a cells without OPE treatment. Cells were also treated with 10% ethanol as a positive control. No significant loss of viability following the 24-h incubation period was observed from any of the OPEs in the 1–10 μM concentration range. For ThT, no loss of viability was observed at the low concentrations of 1 and 5 μM. However, at the high tissue staining concentration of 1.57 mM ThT, there was a complete loss of cell viability. Cationic phenylene ethynylenes have been shown to exhibit selective antimicrobial activity through binding and disruption of anionic microbial cell walls and cell membranes. Microbial toxicity is enhanced with light irradiation, as OPEs are also photosensitizers that generate singlet oxygens in the presence of light [70,71]. Unlike bacterial cells that possess an overall negative charge at neutral pH, mammalian cell membranes are largely composed of zwitterionic lipids, which reduces the electrostatic interactions between the cationic phenylene ethynylenes and the cell surface [72,73]. Our results that the OPEs are not cytotoxic toward the N2a cells are consistent with previous studies that found OPEs to be nontoxic toward other mammalian cells while exerting antimicrobial and antiviral activities [74,75,76]. We also showed in a recent study that OPE_1_^2−^ is not toxic toward SHSY-5Y human neuroblastoma cells [77].

## 4. Conclusions

As our population ages, there is an increased need to develop diagnostically relevant tools to aid in the detection and treatment of neurodegenerative diseases such as AD. In previous work, we found that small OPEs selectively bind to and detect fibrillar and pre-fibrillar aggregates of amyloid proteins in vitro. In this study, we evaluated and characterized the capacity of two OPEs as ex vivo amyloid sensors. Brain tissues from wildtype (C57BL/6J, Fisher 344) and transgenic AD (rTg4510, TgF344-AD) mice and rats along with human brain tissue from non-demented healthy controls and FTD were incubated with two OPEs and characterized for staining patterns. The anionic OPE_1_^2−^ selectively stained tau NFTs in mouse and human tissue at 5 µM. The cationic OPE_2_^4+^ at 5 µM showed some non-specific binding in all brain sections, but at a lower concentration of 0.5 µM, this problem was resolved and the sensor displayed selectivity for staining tau NFTs. OPE_1_^2−^ staining co-localized with the binding of the phosphorylated tau antibody AT180 and ThT in mouse and human sections, confirming that the OPE-stained tau NFTs. Furthermore, quantitative analysis showed that OPE_1_^2−^ is better at staining NFTs than AT180. In the rat AD model, OPE_2_^4+^ displayed selectivity for Aβ plaques at 5 µM with minimal background fluorescence in the Fisher-344 wildtype sections. OPE_2_^4+^ co-localized with Aβ specific antibody 4G8 on the same plaques but stained the core, while the antibody stained the more diffuse morphology. In the rat sections, we did not observe consistent OPE_1_^2−^ staining of plaques. The rat brain sections did not have tau pathology for the OPEs to detect; therefore, we did not see NFT staining in rats. Lastly, we evaluated the OPEs’ toxicity toward N2a cells using the MTT assay. Neither OPE caused a significant loss of cell viability at concentrations up to 10 µM. In comparison, ThT at a tissue staining concentration of 1.56 mM caused a complete loss of viability of the N2a cells. Our results thus show that OPEs are effective ex vivo markers for the selective staining of tau NFTs and Aβ amyloid plaques at concentrations orders of magnitude lower than ThT. This work paves the way for developing these novel sensors for in vivo diagnosis and tracking of disease progression in numerous neurodegenerative diseases. The versatility of the OPEs opens the door not only for the detection of neurodegeneration but also for the detection of general amyloidogenic disorders.

## Figures and Tables

**Table 1:** Amyloid Detection Factor (ADF) values of OPE_1_^2−^ and OPE_2_^4+^ for mouse and human-derived PHFs.

	Mouse PHF	Human PHF
**OPE_1_^2−^**	2.74	2.62
**OPE_2_^4+^**	−0.21	0.55

## Data Availability

The data presented in this study are available on request from the corresponding authors.

## Supplementary Materials

The following supporting information can be downloaded at: https://www.mdpi.com/article/10.3390/bios13020151/s1, Figure S1: Images of 6-month-old C57BL/6J WT, 9-month-old rTg4510, and human FTD brain sections stained with 5 µM and 1.57 mM Thioflavin T (ThT); Figure S2: Confocal images of 9-month-old C57BL/6J WT and human non-demented healthy control brain sections stained with 5 µM and 0.5 µM OPEs; Figure S3: Confocal images of single-stained brain sections from 10-month-old Fischer 344 (wildtype) and transgenic TgF344-AD rats. Sections were stained with Aβ-specific antibody 4G8, tau-specific antibody AT180, 5 µM ThT, and 1.57 mM ThT.
