# Supplementary material for: Selective In Vitro and Ex Vivo Staining of Brain Neurofibrillary Tangles and Amyloid Plaques by Novel Ethylene Ethynylene-Based Optical Sensors
Source: Biosensors (Basel). 2023 Jan 18;13(2):151. doi: 10.3390/bios13020151 (PMC9953543; doi:10.3390/bios13020151)
Supplement: Supplementary file 1 [file biosensors-13-00151-s001.zip › biosensors-2099424-supplementary.pdf]

Supplemental Materials

Selective In Vitro and Ex Vivo Staining of Brain Neurofibrillary Tangles and Amyloid Plaques by Novel Ethylene Ethynylene-Based Optical Sensors

Florencia A. Monge [1,2], Adeline M. Fanni [1,2], Patrick L. Donabedian [2,3], Jonathan Hulse [4], Nicole M. Maphis [4,5], Shanya Jiang [5,6], Tia N. Donaldson [7], Benjamin J. Clark [7], David G. Whitten [2,8], Kiran Bhaskar [5,*] and Eva Y. Chi [2,7,*]

1 Biomedical Engineering Graduate Program, University of New Mexico, Albuquerque, NM 87131, USA
2 Center for Biomedical Engineering, University of New Mexico, Albuquerque, NM 87131, USA
3 Nanoscience and Microsystems Engineering Graduate Program, University of New Mexico, Albuquerque, NM 87131, USA
4 Molecular Genetics and Microbiology, University of New Mexico, Albuquerque, NM 87131, USA
5 Department of Neuroscience, University of New Mexico, Albuquerque, NM 87131, USA
6 Sartorius, Bohemia, NY 11716, USA
7 Department of Psychology, University of New Mexico, Albuquerque, NM 87131, USA
8 Department of Chemical and Biological Engineering, University of New Mexico, Albuquerque, NM 87131, USA
* Correspondence: KBhaskar@salud.unm.edu (K.B.); evachi@unm.edu (E.Y.C.);
Tel.: +1-505-272-1230 (K.B.); +1-505-277-2263 (E.Y.C.)

Keywords: biosensors; neurofibrillary tangles; amyloid plaques; dyes; optical imaging; fluorescence imaging; tau; paired helical filaments

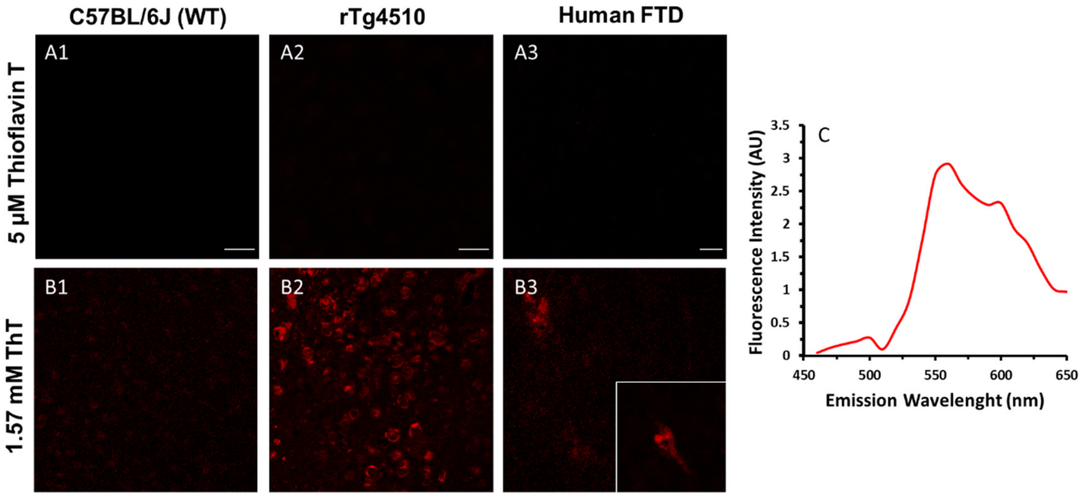

Figure S1. Images of 6-month-old C57BL/6J WT, 9-month-old rTg4510, and human FTD brain sections stained at 5 µM (A1, A2, and A3) and 1.57 mM (B1, B2, B3) ThT. The low concentration of 5 µM matches that used for OPE staining and the high 1.57 mM is a concentration commonly used in published protocols. Scale bars = 25 µm. The emission spectra of single stained ThT stained 9-month old rTg4510 section exited at 458nm (C).

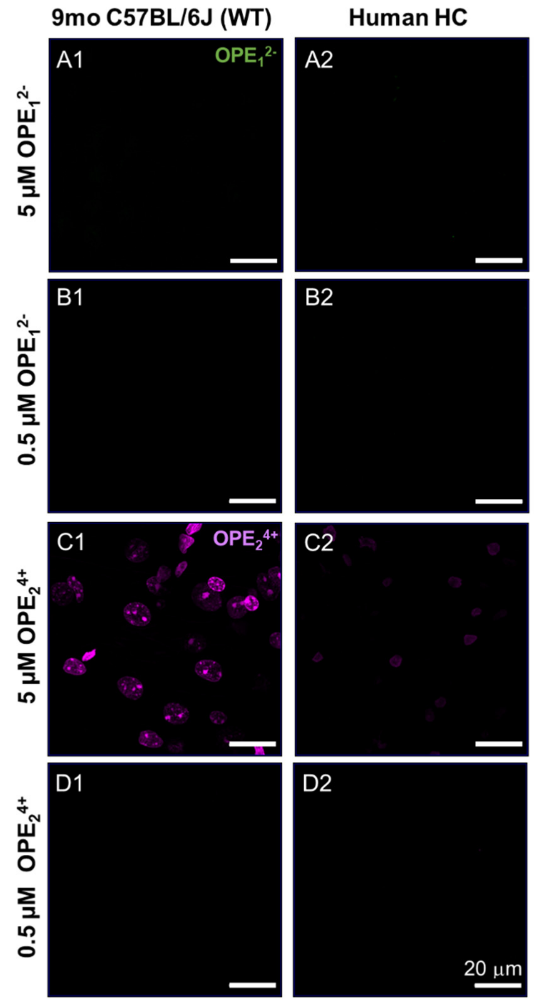

Figure S2. Confocal microscopy images of *ex vivo* OPE stained cortex brain sections from 9-month old non-transgenic C57BL/6J mice (left column) and non-demented human healthy control (right column). At 5 µM (A1-2) and 0.5 µM (B1-2), OPE_1^{2-} showed no fluorescence in either the aged non-transgenic mouse or healthy human control sections. At 5 µM (C1-2), OPE_2^{4+} showed non-NFT binding in both the aged non-transgenic mouse and human healthy control sections. At 0.5 µM, this non-specific binding was significantly reduced in both the mouse and human sections. Scale bars = 20 µm. Images were taken using a Zeiss LSM 900 confocal microscope with excitation using a 405 nm laser line and emission collected from 400 to 600 nm.

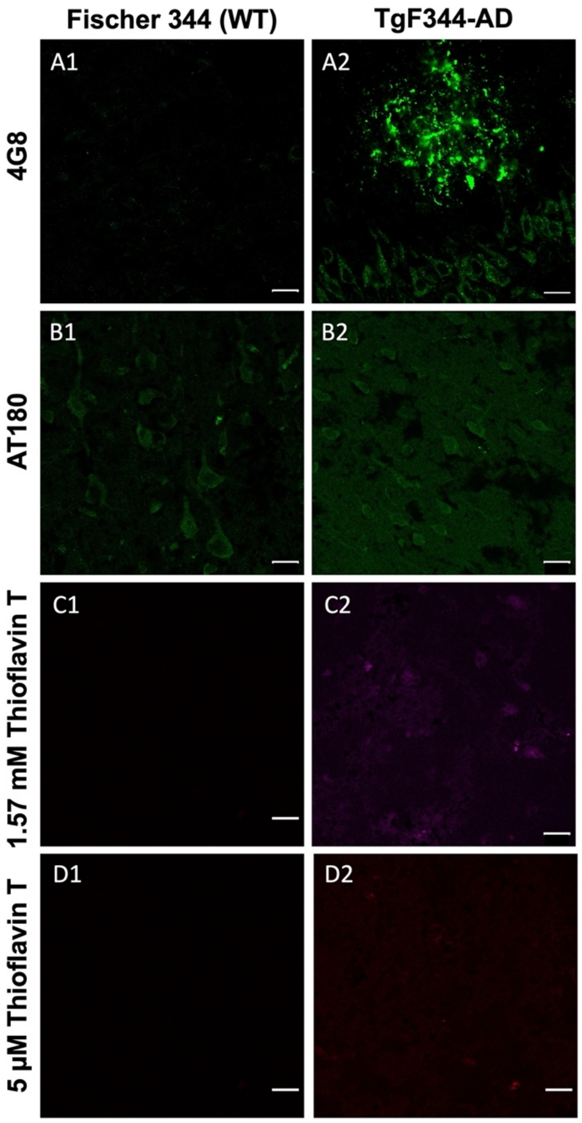

Figure S3. Confocal images of single stained brain sections from 10-month-old Fischer 344 (wildtype) and transgenic TgF344-AD rats. Scale bars = 25 µm. Sections were stained with Aβ-specific antibody 4G8 (A1 and A2), tau specific antibody AT180 (B1 and B2), 5 µM ThT (C1-C2), and 1.57 mM ThT (D1-D2). Only the 4G8 antibody stain detected plaques in the TgF344AD in this age group. Using a Leica TCS SP8 confocal microscope, the ThT stained sections were imaged using excitation from the 458 nm Argon line and emission was collected from 460 to 650 nm. The same laser intensity was used for both ThT concentrations. The antibody-stained sections were co-stained with AF555 for visualization; the sections were excited with a tunable white light laser at 555 nm and emission was collected 560 to 700 nm.
